# Functional Analysis of the Drosophila Embryonic Germ Cell Transcriptome by RNA Interference

**DOI:** 10.1371/journal.pone.0098579

**Published:** 2014-06-04

**Authors:** Ferenc Jankovics, László Henn, Ágnes Bujna, Péter Vilmos, Kerstin Spirohn, Michael Boutros, Miklós Erdélyi

**Affiliations:** 1 Institute of Genetics, Biological Research Centre of the Hungarian Academy of Sciences, Szeged, Hungary; 2 German Cancer Research Center (DKFZ) and Heidelberg University, Division Signaling and Functional Genomics, Heidelberg, Germany; University College London, United Kingdom

## Abstract

In *Drosophila melanogaster*, primordial germ cells are specified at the posterior pole of the very early embryo. This process is regulated by the posterior localized germ plasm that contains a large number of RNAs of maternal origin. Transcription in the primordial germ cells is actively down-regulated until germ cell fate is established. Bulk expression of the zygotic genes commences concomitantly with the degradation of the maternal transcripts. Thus, during embryogenesis, maternally provided and zygotically transcribed mRNAs determine germ cell development collectively. In an effort to identify novel genes involved in the regulation of germ cell behavior, we carried out a large-scale RNAi screen targeting both maternal and zygotic components of the embryonic germ line transcriptome. We identified 48 genes necessary for distinct stages in germ cell development. We found *pebble* and *fascetto* to be essential for germ cell migration and germ cell division, respectively. Our data uncover a previously unanticipated role of *mei-P26* in maintenance of embryonic germ cell fate. We also performed systematic co-RNAi experiments, through which we found a low rate of functional redundancy among homologous gene pairs. As our data indicate a high degree of evolutionary conservation in genetic regulation of germ cell development, they are likely to provide valuable insights into the biology of the germ line in general.

## Introduction

The fruit fly, *Drosophila melanogaster,* provides a powerful experimental model system for the genetic dissection and *in vivo* analysis of germ cell totipotency. At the onset of *Drosophila* embryogenesis, primordial germ cells (PGCs) bud at the posterior pole of the syncytial embryo. By their formation, PGCs incorporate a specialized cytoplasm, the so-called germ plasm, which contains maternally provided transcripts and proteins [1]. Once established, PGCs segregate from the somatic cell line. At this stage, maternally provided mRNAs and proteins regulate the maintenance of the undifferentiated PGC's state. PGC-enriched maternal transcripts and proteins involve stem cell proliferation regulators, such as *mei-P26*, reflecting the importance of proliferation control in PGC development [2]. After their formation, PGCs attach to the underlying midgut anlage and are passively transported into the midgut primordium. In the midgut, most of the maternal mRNAs are rapidly degraded and zygotic transcription from the PGC genome is initiated. The zygotically transcribed genes regulate the subsequent chemotactic migration of the PGCs in the body cavity, which involves crossing the midgut epithelium, splitting the PGCs into two cell groups and their migration to the mesoderm. Here, PGCs coalesce with the mesodermal somatic gonadal precursor cells (SGPs) and form a pair of compact gonads [3]. At this time, sexual identity of the germ cells is determined, and embryonic testes and ovaries are established [4].

In the embryonic testes, a small subset of male PGCs give rise to male germ line stem cells (GSCs) and populate the stem cell niche [5]. In the female larva, PGCs first undergo three rounds of mitosis and form larval ovaries composed of approximately hundred undifferentiated PGCs and several somatic cell types [6]. At the third larval stage, however, a subset of PGCs gives rise to GSCs and populates the ovarian germ line stem cell niche [7]. In both sexes, GSCs are maintained by a combination of regulatory processes which involve various genetic and epigenetic processes, such as chromatin remodeling, interaction of signaling pathways or miRNA mediated post-transcriptional gene silencing [8], [9]. Transplantation experiments have revealed that adult GSC-like tumor cells can function as embryonic PGCs, suggesting that common mechanisms exist to maintain PGC and GSC fates [10]. Genetic analysis has demonstrated that shared mechanisms ensuring maintenance of PGCs and GSCs are regulated by the same set of genes, including *nanos (nos)* and *pumilio (pum)* [11].

In both sexes, GSCs undergo permanent asymmetric mitotic cycles, producing another GSC and a transit amplifying cell differentiating into highly specialized oocytes and sperms. Although they are specialized, upon fertilization gametes produce the totipotent zygote, which possesses the ability to generate all types of tissues. Intriguingly, this obvious capacity of the mature germ cells to maintain totipotency is regulated by similar conserved mechanisms which ensure the undifferentiated state of stem cells or malignant tumor cells [12], [13]. In a wide variety of animals, these extensive similarities among stem cells and germ line cells seem to be ensured by the same set of genes, including *nos*, *vasa, pum* and *piwi* [14], [15]. Identification of additional genes involved in germ cell development may contribute to a better understanding of regulatory processes that maintain the totipotency of the germ line.

In the last decade, numerous large-scale gene expression studies have identified a plethora of genes expressed at distinct developmental stages in the germ line [2], [16]–[18]. However, our knowledge of the specific function of these genes is still very limited. Therefore, we applied a large-scale RNAi-based screen to systematically investigate the molecular mechanisms underlying germ cell development. Here, we present the identification of 48 genes involved in various steps of germ cell development. A subset of the genes regulates developmental stage-specific tasks of germ cell behavior, such as cell division, chemotactic migration or coalescence with somatic cells. Another subset is required for survival of the embryonic and larval PGCs, indicating a general requirement of these genes in the germ line. The majority of the identified genes encode conserved proteins with diverse molecular functions, several of which have not been implicated previously in regulating germ line function.

## Results

### Identification of genes required for distinct stages of germ cell development

For the systematic analysis of the embryonic germ line transcriptome, first we screened publicly available databases for germ line-localized transcripts. We used the *in situ* hybridization data of the BDGP and fly-FISH databases and microarray data on separated germ cells to assemble a list of genes expressed in the germ-line at any stage of embryonic development [16]–[18]. In this way, 502 genes were selected whose transcripts are present or highly enriched in the germ plasm or expressed in the germ cells at various stages throughout embryonic development (Table S1). Thus, the selected transcripts involve maternally provided as well as zygotically transcribed mRNAs.

**Figure 1:**
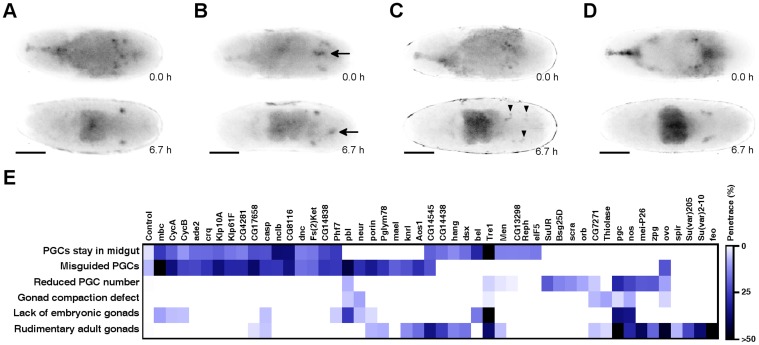
RNAi screen reveals genes required for embryonic germ cell development. (A–D) Frames from movie sequences show germ-cell development of wild type and dsRNA-injected embryos with abnormal germ cell development. Embryos express EGFP in the germ cells. All embryos are shown in dorsal view with anterior to the left. The scale bar represents 50 µm. (A) Control embryo injected with buffer. (B–D) Examples for various germ-line defects. (B) Embryo injected with *CG8116* dsRNA. Arrows indicate germ cells stuck in the midgut. (C) Embryo injected with *pbl* dsRNA. PGCs are scattered in the body cavity (arrowheads), their number is reduced and no embryonic gonads were formed. (D) Embryo injected with *neur* dsRNA shows gonad compaction defects. (E) Heat map representation of the RNAi phenotypes following hierarchical clustering. Color code represents penetrance of the phenotypic categories.

To investigate the function of the germ line transcriptome, we performed a large-scale RNAi-based screen. The selected genes were silenced by microinjecting dsRNAs specific to each of the 502 genes into syncytial embryos (Table S1) [19], [20]. In this experimental setup, the selected genes were silenced both in the embryonic germ line and in the soma thereby revealing their germ cell-autonomous and non-autonomous effect on germ line development. Loss-of-function RNAi phenotypes were recorded at two distinct developmental stages: during embryogenesis and in adult flies. The primary phenotypic analysis was performed *in vivo* by fluorescent time-lapse microscopy on embryos expressing Moesin:GFP in the germ line [21]. Germ cell development in dsRNA-treated embryos was recorded throughout embryogenesis and the movies were analyzed by visual inspection (Figure 1A–D, Movie S1). During the course of the study, movies from more than 110,000 embryos were acquired and annotated. When the penetrance of a mutant phenotype exceeded twice that of the control in two independent experiments, the gene was identified as a true positive hit.

Since detection of the phenotypes was performed by *in vivo* imaging, it was also possible to analyze germ cell phenotypes at later developmental stages. Thus, as a secondary screen, the examined embryos were permitted to develop until adulthood and their adult gonads were assayed for phenotypic defects. For this purpose, over 11,000 adults were hand dissected and analyzed. In both screens each detectable phenotype was confirmed by at least two independent experiments. In this way, the silencing of 48 genes which represent 9.6% of the analyzed germ line transcriptome caused abnormal phenotypes at various stages of germ cell development. To test the reliability of our screening approach, 26 randomly selected genes were included in the study. No abnormal germ cell development was detected in these control experiments, indicating that the examined subset of genes is enriched in genes required for germ line development (Table S2).

To avoid off-target effects, new dsRNAs targeting other regions of the 48 newly identified genes were synthesized and subsequently microinjected in two independent experiments (Table S3). Time-lapse microscopy of the embryos and dissection of the adults reproducibly resulted in the expected RNAi phenotypes by all of the 48 genes, confirming the reliability of our approach. In summary, the application of our screening strategy enabled the identification of 48 genes that play a role in germ cell development. (Figure 1E, Table S2).

### Phenotypic profiling of complex germ line phenotypes

Inspection of a large number of movies revealed that silencing of most of the genes results in complex germ cell phenotypes. In addition, expressivity of the germ cell defects varied from embryo to embryo, rendering simple phenotypic classification unfeasible. Therefore, each movie was re-evaluated and the complex germ cell phenotypes were split into five defect categories: reduced germ cell number, misguided germ cells, germ cells stuck in the midgut, and absence or abnormal compaction of the embryonic gonads. Rudimentary adult gonads were also considered an additional defect category (Figure 1E). To ensure consistent evaluation, the final annotation of the phenotypes was performed by one scientist (LH). The observed germ cell defects were organized into a database, and the penetrance of the phenotypic abnormalities was determined (Table S2).

The earliest defect we were able to detect was the delay in or lack of transepithelial migration of the germ cells through the midgut epithelium. As a consequence, some PGCs were stuck in the midgut, where they persisted until the end of embryogenesis. In some cases (silencing of *Tre1*, *bel*), all PGCs were trapped in the midgut in most of the dsRNA-treated embryos. Since *Tre1* has been shown to affect transepithelial migration of PGCs, the recovery of this true positive hit in our candidate list is an additional confirmation of the reliability of our screening approach, whereas *bel* is a novel factor regulating transepithelial migration [22]. In our screen, transepithelial migration defects frequently coincided with later defects in germ cell migration, suggesting that correct timing of crossing the midgut epithelium is required for later events.

A common developmental defect was the abnormal migration of the PGCs towards the somatic gonad components (*pbl, mbc, Klp10A, Klp61F* and *CG8116*). We only considered cases as positive where more than one PGCs/embryo were misguided and scattered in the body cavity.

Silencing of many genes resulted mainly in progressive loss of germ cells, indicating a role for these genes in germ cell survival. In less severe cases, some PGCs survived and were able to set up the embryonic gonads, which contained fewer germ cells than wild-type gonads (*SuUR*, *orb*, *scra*). If these embryos developed into adults, this abnormality was compensated and normal adult gonads were established. In more severe cases this germ cell loss was associated with additional later defects, such as failure in gonad compaction or rudimentary adult gonads (*CG7271*, *mei*-*P26*, *zpg*). In the most severe cases, such as silencing of *nos* and *pgc*, in most of the dsRNA-treated embryos, all germ cells were lost and germ cell-less embryonic and adult gonads were formed.

The latest embryonic phenotype observed was the failure of gonad coalescence. In these embryos, the PGCs migrated properly and appeared to reach the SGPs; however, only loosely compacted gonads were formed. We could also observe that some PGCs detached from the SGPs and scattered close to the gonad (*mei*-*P26*, *neur*).

Silencing of 23 genes was found to lead to formation of rudimentary gonads at adult stage. This group of 23 genes can be separated into two subgroups based on their effect on embryonic PGC development. Silencing of 19 members of the first subgroup also caused various abnormalities in embryonic germ cell development, such as fewer germ cells or misguided germ cells. However, silencing of the second subgroup, consisting of the remaining 4 genes, did not cause any germ line defect in the embryo, suggesting a requirement of these genes after embryonic gonad coalescence at the larval stages.

At this stage of the analysis, we did not discriminate between completely germ cell-less gonads and gonads with abnormal cysts or egg chambers indicating early germ line survival defects and germ cell differentiation defects, respectively. To discriminate between these two possibilities of the rudimentary adult gonad phenotype and to test their germ line dependence, genes were silenced using germ-line specific expression of inducible transgenic shRNAs. Transgenic shRNA constructs were available for 18 out of the 23 genes. Germ-line specific silencing of the zygotic expression of seven genes (*Aos1*, *feo*, *mei*-*P26*, *Su(var)2-10*, *Su(var)205*, *zpg, hang*) resulted in severe adult germ cell defects, demonstrating a functional requirement of these genes in the germ line (Table S4). Except for *hang*, in the gonads silenced for these genes, some of the rudimentary ovaries completely lacked germ cells, indicating a role of these genes in the survival of PGCs or GSCs (Figure S1). Inducible silencing of three additional genes of this category (*nos*, *dsx*, *Tre1*) resulted in completely germ cell-less adult gonads when the shRNAs were provided maternally, confirming a role of these genes in the early stages of embryonic germ cell development (Table S4).

The identified genes were ordered by hierarchical clustering based on the penetrance of the respective mutant defect categories. To obtain a global overview of the complex loss-of-function RNAi phenotypes, the penetrance of the defect categories was visualized on a heat map (Figure 1E). Gene Ontology analysis of the genes showed that silencing of genes with similar biological functions frequently resulted in similar complex phenotypes For example, the gene group characterized mainly by a misguided germ cell phenotype contained two kinesin and two cyclin genes, indicating an importance of cell division and cell cycle regulation in early germ cell development. In addition, chromatin regulators, such as *Su(var)2-10* or *Su(var)205*, clustered to the gene group characterized by rudimentary adult gonads without embryonic germ cell phenotype suggesting a fundamental function of epigenetic regulation in germ cell development after the embryonic stage.

### Bioinformatic analysis of the identified genes indicates evolutional and functional conservation

Gene Ontology annotation of the identified *Drosophila* genes showed that 19 of them, representing 39.6% of our hits, have been annotated previously with a function in gamete generation, whereas for the remaining 61.4% of the identified genes our study provides the first functional link to germ cell development (Table S5). Importantly, for ten genes of our candidate list there was no data available previously about their biological function (*ade2, Bsg25D, CG4281, CG7271, CG8116, CG14438, CG14545, CG14838, CG17658, Pglym78*). Through database searches for orthologs of the identified hits in other model organisms, such as the yeast *Saccharomyces cerevisiae*, the nematode *Caenorhabditis elegans* or the mouse, the majority of our genes (37/48) were found to be conserved evolutionarily. Gene Ontology annotations of the orthologous genes were collected and analyzed. Out of the 37 orthologs, 13 have also been reported to be involved in germ cell development in other species, suggesting a functional conservation of genetic elements regulating at least some processes in germ cell development.

### Systematic co-silencing of homologous germ line transcripts revealed low level of functional redundancy

RNAi has been shown to be an ideal method for the simultaneous silencing of genes to systematically reveal genetic interactions. Therefore, we performed a large-scale combinatorial gene silencing to detect genes with redundant function in germ cell development. To target multiple genes simultaneously in a high-throughput setting, we established and silenced pairs of homologous genes from the 502 germ line enriched transcripts (Table S6). Using the Ensemble database, we identified 24 pairs of paralogous genes. In addition, we also set up a pair-wise combination of genes with high overall sequence homology (111 pairs) or sharing identical functional domains (272 pairs). In this way, altogether 384 gene pairs were established and subjected to co-RNAi and the germ cell phenotypes were detected in embryos and in adults. To identify functional redundancy between the gene pairs, the RNAi phenotype of each gene alone was compared to the phenotype resulting from silencing both genes simultaneously.

**Figure 2:**
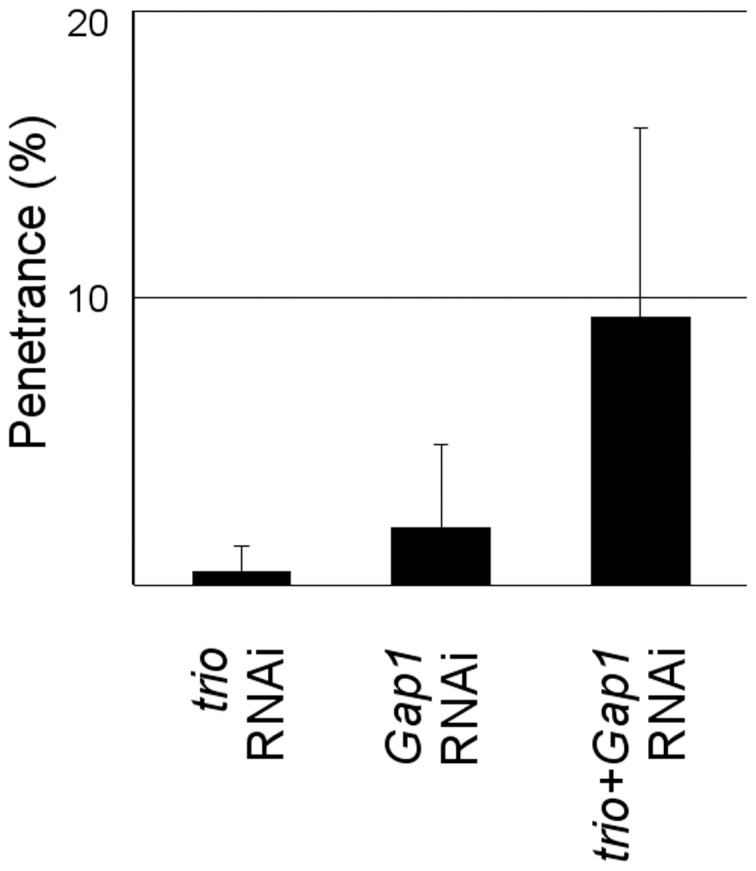
Systematic simultaneous silencing reveals genetic interaction between *trio* and *Gap1*. Graph showing the quantification of the genetic interaction between *trio* and *Gap1*. Co-silencing of *trio* and *Gap1* increased the penetrance of the gonad formation defects induced by silencing of the genes separately (T-test, p<0.05). Bars represent the averaged penetrance of eight individual experiments. Error bars show standard deviation.

Co-silencing of two pairs increased the penetrance of the germ cell defects induced by silencing of the genes separately. For these pairs, new dsRNAs targeting other regions of the interacting genes were synthesized and the simultaneous silencing was repeated. We found that the co-silencing of one gene pair resulted in a reproducible synthetic RNAi phenotype indicating a low level of functional redundancy among the early germ cell transcriptome. Simultaneous silencing of *trio* and *Gap1* genes increased the penetrance of one defect category, i.e. loss of embryonic gonads (Figure 2). Trio and Gap1 regulate the activity of the small GTPases Rho and Ras, respectively. Identification of these genes in our screen indicates the importance of proper GTPase activity in the PGCs.

### Confirmation of RNAi phenotypes by means of genetic interaction

To confirm the involvement of the identified genes in germ cell development, their loss of function alleles were collected and tested for dominant genetic interaction with a sensitized genetic background. The sensitized background magnifies the effect of the loss of function alleles on germ cell development, thus enables the analysis of even lethal alleles of the identified pleiotropic genes in a heterozygous animal. The sensitized females were heterozygous for mutations in the *staufen, oskar* and *TropomyosinII* genes causing a reduction of the germ plasm in the developing oocytes. Reduction of the maternal germ plasm decreased the PGC number in the progenies from the wild type of 22 to 11 which in turn resulted in a 10% penetrant adult germ cell-less phenotype. Females bearing one copy of the *staufen^D3^*, *oskar^54^* and *tropomiosinII^eg9^* alleles and one copy of a loss of function allele of the gene of interest were generated and their progenies were scored for germ cell-less gonads [23]. 87 alleles of 45 genes were tested, and 42 alleles of 31 genes were found to dominantly enhance the penetrance of the germ cell-less phenotype caused by the sensitized genetic background (Table S7). Dominant genetic interactions indicate an important role of the genes in germ cell development. Therefore, three genes (*pebble*, *fascetto*, *mei-P26*), representing various phenotypic groups were selected and their functions were further analyzed in the germ line.

### *Pebble* (*pbl*) has multiple roles in embryonic gonad formation

**Figure 3:**
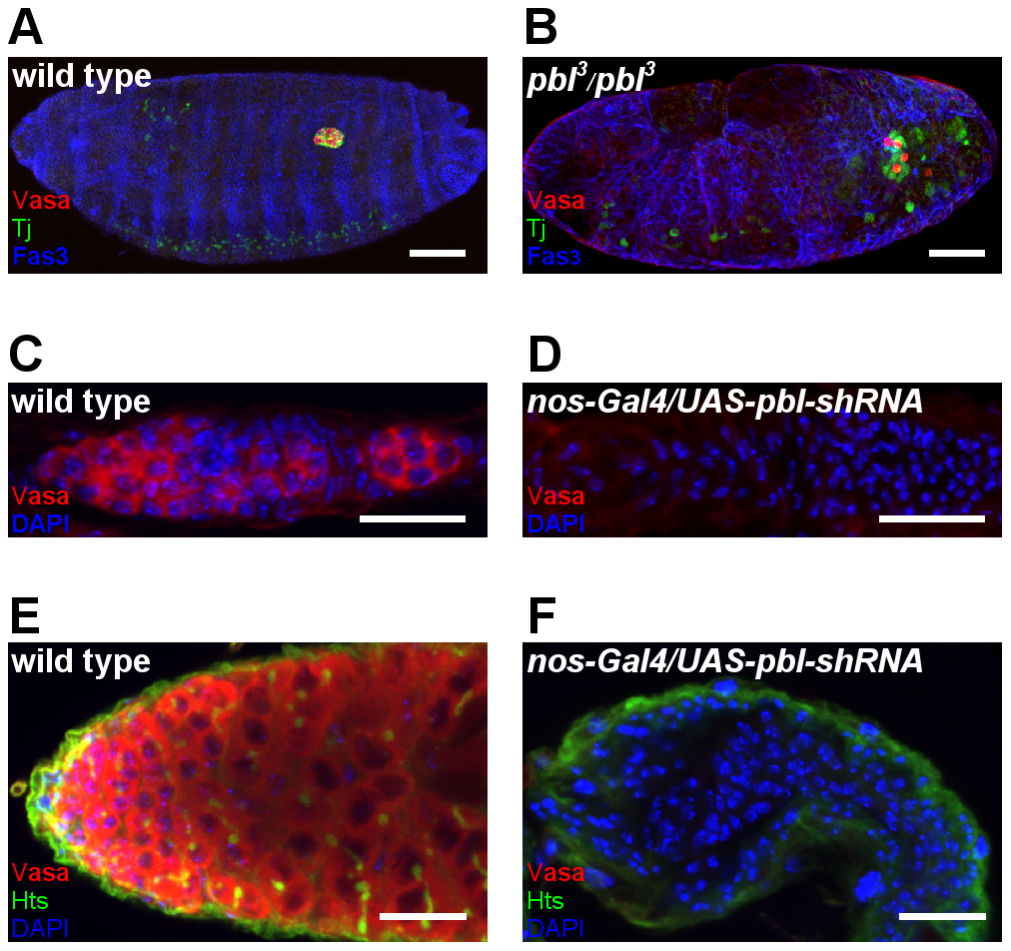
*Pbl* affects germ-cell development. (A,B) Immunostaining of a wild type (A) and a homozygous *pbl^3^* mutant embryo (B). *Pbl* mutants have fewer germ cells, misguided germ cells and abnormally compacted gonads. Vasa staining labels germ cells (red), Tj staining labels the somatic gonad precursor cells (green). Week Tj expression is also detectable in the cenrtral nervous system. The outline of the embryos is marked by Fas3 staining (blue). Scale bar represents 50 µm. (C–F) Immunofluorescence images of adult gonads. Wild type ovariole (C) and testis (E) contain high number of developing germ cells. *Nos-Gal4-VP16/UAS-pbl-shRNA* (TRiP.GL01092) ovariole (D) and testis (F) lack germ cells. Vasa staining labels germ cells (red), DAPI labels the nuclei (blue). Anterior is to the left. Scale bar represents 20 µm.

Silencing of *pbl* resulted in severe defects in embryonic germ cell migration and gonad formation. *Pbl* encodes for a multifunctional RhoGEF involved in the regulation of Rho and Rac GTPases and has been shown to be involved in various biological processes, such as cell division and cell migration. In addition to the defects in morphogenesis, abnormal germ cell development was detected in the *pbl* mutant embryos. Consistent with the data obtained by *pbl* silencing, embryos homozygous for the *pbl^3^* mutation had fewer germ cells, misguided germ cells and abnormal gonad compaction (Figure 1C, Figure 3A-B). In the *pbl* mutant embryos, instead of the wild type 0.2±0.4 (n = 60) PGCs, 2.3±1.7 (n = 23) PGCs were misguided. In the *pbl* mutant gonads, instead of the wild type 10.5±1.5 PGCs (n = 60), only 2.7±2.7 (n = 23) PGCs were found. Since Pbl protein is ubiquitously expressed in the embryos, this phenotype can be a consequence of loss of *pbl* function either in the germ cells or in the SGP cells. To distinguish between the two possibilities, the development of the somatic gonad components was examined by immunostaining of Traffic jam (Tj), a transcription factor expressed in the somatic cells of the embryonic gonads. In the *pbl* mutant embryonic gonads, abnormal distribution of somatic cells was detected, indicating that *pbl* might have multiple roles in gonad formation by affecting both SGP and PGC development.

To further investigate the role of *pbl* in germ cell development in a germ-line specific manner, *pbl* was silenced by *nos-Gal4* driven germ-line specific expression of *pbl-shRNA*. In *pbl* silenced animals, germ cells were lost and germ cell-less adult gonads were formed, indicating a specific requirement of *pbl* in the germ line (Figure 3C–F).

### *Fascetto* (*feo*) is required for PGC division

**Figure 4:**
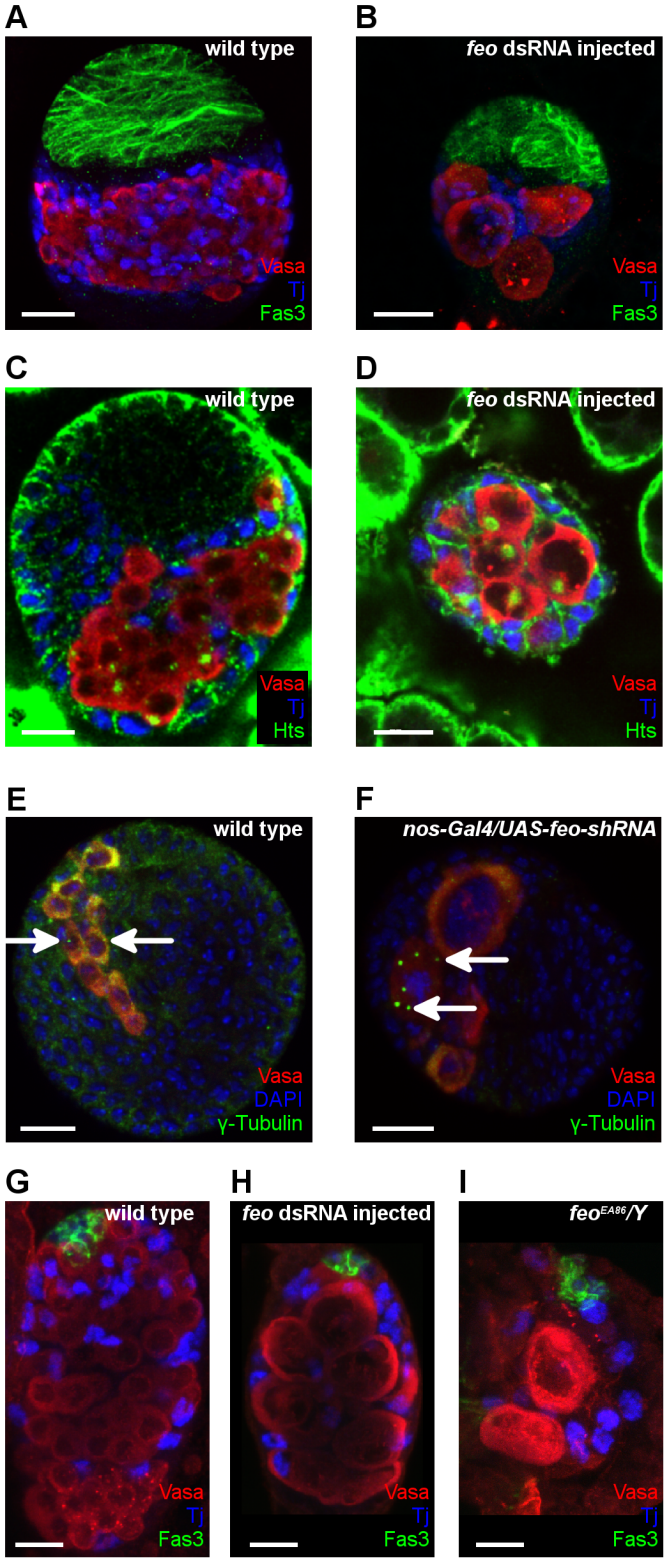
*Feo* is required for mitosis of larval germ cells. (A–D) Immunofluorescence images of third stage larval ovaries. Ovaries of larvae injected with *feo* dsRNA are rudimentary and contain fewer but larger germ cells than the wild type suggesting that the PGCs were unable to undergo mitotic divisions. All ovaries are shown with anterior to the top. Scale bar represents 20 µm. Vasa staining labels the germ cells (red), Tj staining labels the somatic intermingled cells (blue), Fas3 staining labels the anterior somatic cells in (A,B), Hts staining labels the germ-cell specific spherical spectrosomes in (C,D). (E,F) Immunofluorescence images of larval ovaries. (E) Wild-type ovary. (B) Expression of *feo*-shRNA in the germ line driven by the *nos-Gal4-VP16* driver induces PGCs with multiple centrosomes. Vasa staining labels the germ cells (red), γ-Tubulin staining labels the centrosomes (green), DAPI marks the nuclei (blue). Arrows indicate the centrosomes. Scale bar represents 20 µm. (G–I) Immunofluorescence images of first-stage larval testes. (G) Wild-type control testis. (H,I) Testes of a larva treated with *feo* dsRNA (G) and a *feo^EA86^/Y* mutant (I) contain few, abnormally enlarged germ cells. Vasa staining labels the germ cells (red), Tj staining labels the somatic intermingled cells (blue) and Fas3 labels the hub cells (green). Scale bar represents 10 µm.

*Feo* encodes a conserved microtubule-associated protein involved in the bundling of antiparallel microtubules. Silencing of *feo* by dsRNA microinjection induced no detectable defects until the end of embryogenesis, normal embryonic gonads were formed. However, the *feo*-silenced larval ovaries and testes were rudimentary and contained fewer but larger germ cells than the wild type (Figure 4A–F, Movie S2). During larval development, the size of germ cell nuclei increased with age. In larval PGCs expressing *feo-shRNA*, multiple centrosomes were detected indicating that PGCs have undergone multiple rounds of abnormal mitotic cycles (Figure 4E,F).

The data obtained by *feo* silencing were confirmed by the phenotypic analysis of the loss of function *feo* allele. Embryos carrying the hypomorphic *feo^EA86^* mutation exhibited no defects in embryonic gonad formation, but in the *feo* mutant larvae, the same defects in germ cell division were found as in the silenced larvae (Figure 4G–I). This defect was germ cell autonomous, as indicated by the germ cell phenotypes induced by germ-cell specific silencing of *feo* by the UAS/Gal4 system (Figure 4E–F). In adults, most of the *nos-Gal4*/*UAS-feo-shRNA* ovaries completely lacked germ cells, however, in some ovarioles abnormal GSCs with increased size were found, indicating a role for *feo* in GSC division (Table S4, Figure S1D).

**Figure 5:**
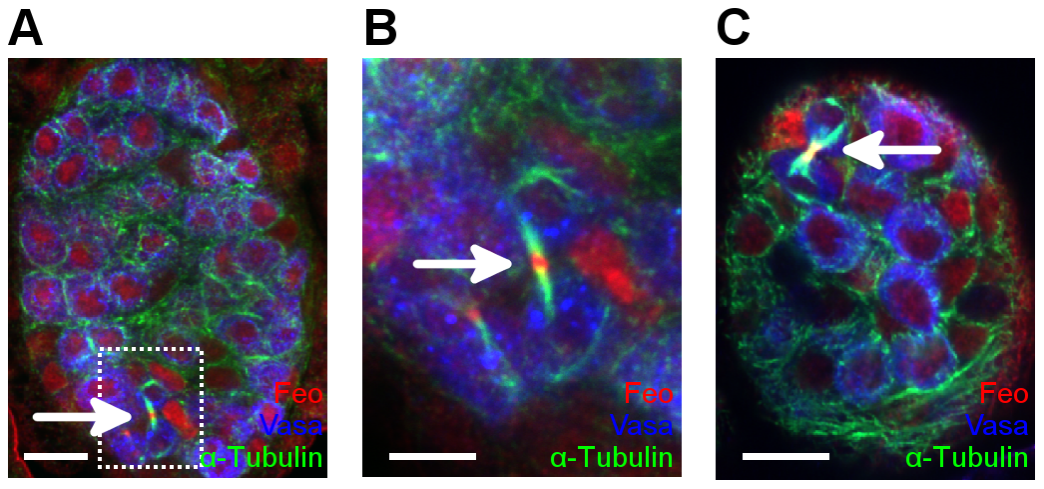
Feo is expressed in the larval germ cells. Localization of Feo in larval gonads. First-stage larval testes (A,B) and ovaries (C) were stained with anti-Feo (red), anti-α-Tubulin (green) and anti-Vasa (blue) antibodies. Arrows indicate the localization of Feo at the spindle midbody in dividing germ cells. (B) Enlargement of the boxed area in (A). Scale bar represents 10 µm in H,J and 5 µm in I.

During embryogenesis, no Feo expression was detectable in the interphase PGCs. After the completion of embryogenesis, germ cells started proliferating in larval gonads. Feo accumulated in the larval germ cells in both sexes, where it localized to the nuclei of the germ cells in interphase. During the mitotic telophase of germ cell division it translocated to the spindle midbody, indicating a role of Feo in the regulation of germ cell mitosis in larval stages (Figure 5A–C).

### *Mei-P26* is expressed in the PGCs and regulates their proliferation

**Figure 6:**
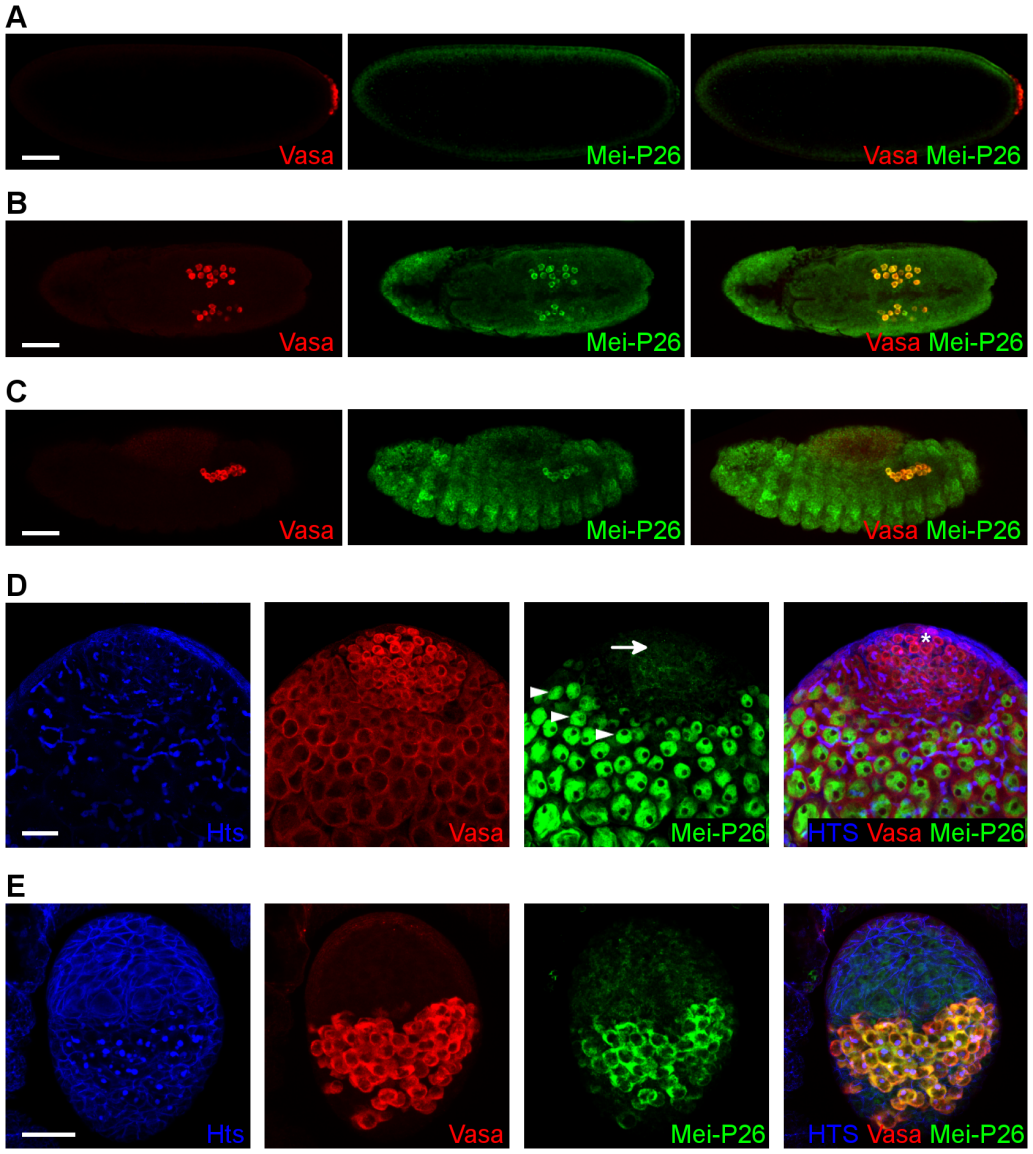
Mei-P26 is expressed in the embryonic and larval PGCs. (A–E) Immunofluorescence staining of embryos (A–C), of a third stage larval testis (D), and of a third stage larval ovary (E). Vasa staining (red) marks the germ cells, Mei-P26 staining (green) indicates the localization of Mei-P26 and Hts staining (blue) labels the fusomes in (D) or the spectrosomes in (E). (A–C) Immunofluorescence images of wild-type embryos. Throughout embryogenesis, Mei-P26 is detectable after formation of the pole cells in the germ line. (A) Lateral view of an embryo at stage 4. (B) Dorsal view of an embryo at stage 11. (C) Lateral view of an embryo at stage 13. Scale bars represent 50 µm. (D) Apical tip of a testis from a third-stage larva. Asterisk indicates the hub cells. Mei-P26 is weekly expressed in the GSCs (arrow) and accumulates in the in the nuclei of differentiating spermatocytes (arrowheads). Scale bar represents 20 µm. (E) Ovary of a wild-type third-stage larva. Mei-P26 accumulates in the germ cells. Anterior is at the top, scale bar represents 20 µm.

Mei-P26 belongs to the conserved TRIM-NHL protein family of translational regulators. To investigate the role of the *mei*-*P26* gene in germ cell development, expression of the Mei-P26 protein was analyzed throughout germ line development by immunostaining (Figure 6). Through *in situ* hybridization, *mei-P26* mRNA has been shown to be expressed throughout the entire embryonic development in the germ line [24]. However, no Mei-P26 protein was detected in the early embryos in the pole plasm. Translation of the *mei*-*P26* transcript starts in the freshly formed PGCs at the posterior pole, where the Mei-P26 protein was found to be cytoplasmic. Mei-P26 persisted in the PGCs until the end of embryogenesis (Figure 6A–C). In larval and early pupal ovaries, Mei-P26 was also expressed in the PGCs (Figure 6E). In larval testes, weak Mei-P26 expression was found in the GSCs. During spermatogonial cyst differentiation, however, a strong Mei-P26 expression was detected in the spermatogonia, where it accumulated in the nuclei (Figure 6D).

**Figure 7:**
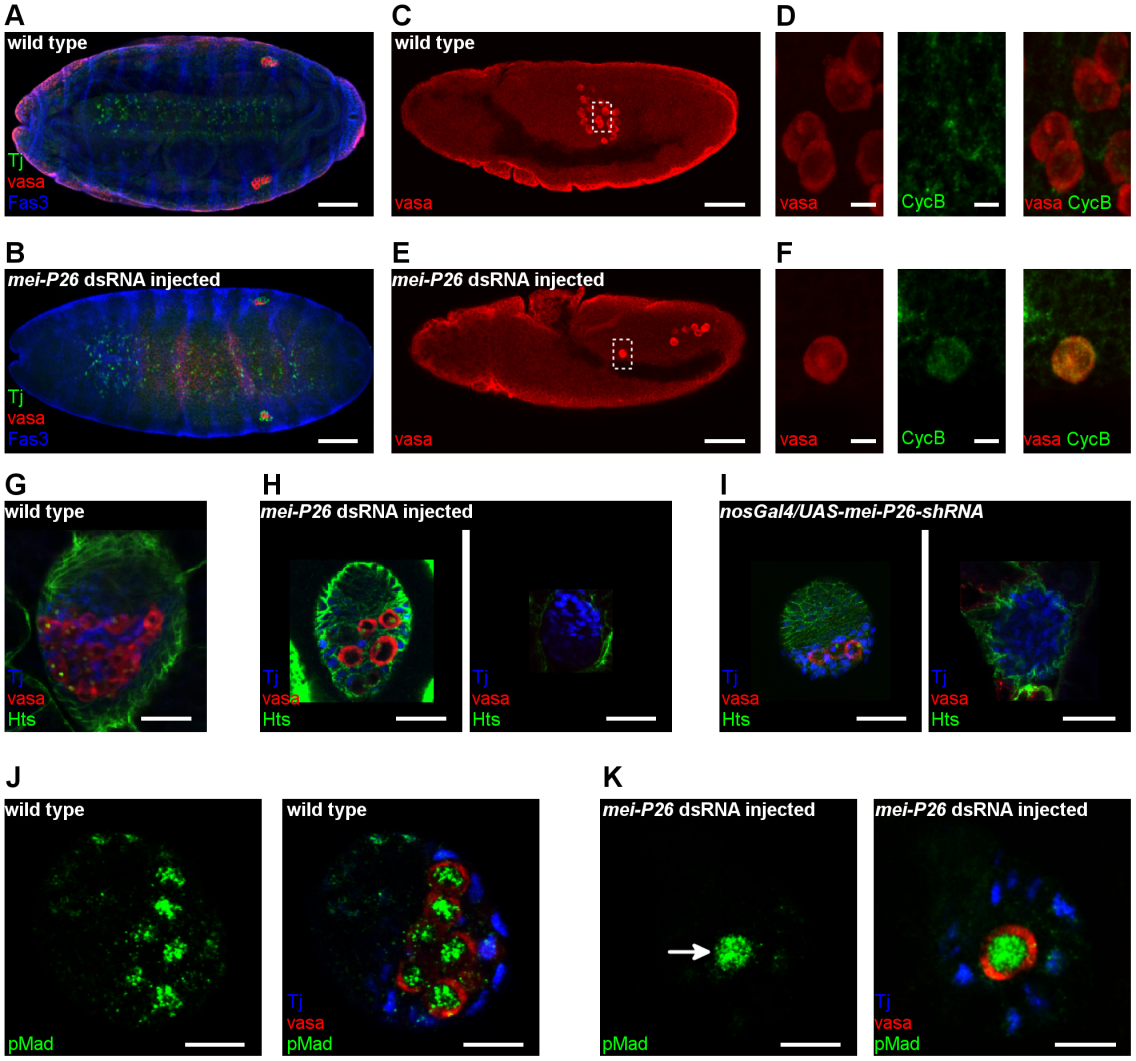
*Mei-P26* regulates PGC development. (A,B) Immunofluorescence images of embryos at stage 16, stained with anti-Vasa (red), anti-Tj (green) and anti-Fas3 (blue) antibodies. (A) Wild-type embryo. (B) Embryo injected with *mei-P26* dsRNA has a reduced number of PGCs in the gonads. Ventral view is shown with anterior to the left. Scale bars represent 50 µm. (C–E) Immunostaining of embryos at stage 11 stained with anti-Vasa (red) and anti-CycB antibodies (green). Lateral view is shown; scale bars represent 50 µm in C,E and 5 µm in D,F. (C) Wild-type control embryos. (D) Enlargement of the boxed region in (C). In the wild-type embryos, germ cells do not express CycB. (E) Embryo injected with *mei-P26* dsRNA. (F) Enlargement of the boxed region in (E). In embryos injected with *mei-P26* dsRNA, germ cells express CycB prematurely. (G–I) Immunofluorescence images of ovaries of third-stage larvae stained with anti-vasa (red), anti-Hts (green) and anti-Tj (blue) antibodies. Scale bars represent 20 µm. (G) Wild-type ovary. (H,I) Larval ovaries with reduced *mei-P26* function have rudimentary ovaries and contain few or no germ cells.(H) Ovaries of larvae treated with *mei-P26* dsRNA. (I) Ovaries of *nos-Gal4-VP16/UAS-mei-P26-shRNA* larvae. (J-K) Immunostaining of ovaries at third larval stage with anti-Vasa (red) and anti-pSmad antibodies (green). DAPI labels the nuclei (blue). (J) Wild-type ovary. (K) Larval ovary treated with *mei-P26* dsRNA. PGCs with reduced *mei-P26* function express the Dpp downstream transducer pMad (arrow). Scale bars represent 10 µm.

A critical role for *mei-P26* has been shown in the adult germ cells; that of an essential regulator of GSC maintenance and cyst proliferation [25]–[27]. However, silencing of *mei-P26* by RNAi induced a much earlier defect in the development of the germ line, indicating a novel role of *mei-P26* in the survival of embryonic PGCs. In embryos treated with *mei*-*P26* dsRNA, loss of PGCs, germ cell migration defects and abnormal gonad formation were detected (Figure 7A,B). This phenotype resembles the *nos* mutants where PGCs adopt somatic cell fate, express CyclinB protein and undergo mitotic division prematurely [28]–[30]. Similar to *nos* mutants, in embryos injected with *mei-P26* dsRNA, PGCs express the CyclinB protein, suggesting a role for *mei-P26* in the maintenance of germ cell fate and suppression of somatic differentiation of embryonic PGC (Figure 7C-F).

In *mei-P26* dsRNA-treated larvae, rudimentary gonads with a reduced number or lack of germ cells were found (Figure 7H). At the adult age, germ cell-less gonads were found (Figure 1, Table S2). This phenotype was germ cell autonomous, as indicated by the identical phenotype induced by germ-line specific silencing of *mei-P26* using shRNA expression in the germ cells (Figure7I, Figure S1G). To explore the requirement for *mei-P26* within larval PGCs, we investigated Dpp signaling and the differentiation state of PGCs. PGCs with impaired Dpp signaling differentiate prematurely into germ line cysts, and instead of a spherical spectrosome, a branched fusome structure appears in the cells [11]. In larval ovaries with reduced *mei-P26* function, all PGCs contained a spherical spectrosome, a cytoplasmic organelle specific to the undifferentiated PGCs and GSCs, indicating that the PGCs did not differentiate prematurely (Figure 7G–I). In addition, PGCs expressed the Dpp downstream transducer phosphorylated-Mad (pMad) indicating that *mei-P26* is not required for Dpp-mediated suppression of PGC differentiation (Figure 6J,K).

## Discussion

In *Drosophila*, PGCs are specified at the posterior pole of the very early embryo by localized RNAs and proteins provided by the oocyte cytoplasm. Transcription in the PGCs is actively down-regulated and the zygotic gene expression does not start before PGCs initiate the transmigration of the midgut epithelium. In this period, exclusively the maternally provided mRNAs and proteins regulate PGC development. In addition, maternally provided transcripts and proteins may also be stable enough to regulate later events of germ cell behavior, such as chemotactic migration, gonadal coalescence and compaction or even GSC specification. This obvious maternal effect makes the identification of genes regulating PGC development challenging. Indeed, exhaustive zygotic screens revealed only *Tre1* as the sole germ cell autonomous gene regulating embryonic germ cell behavior [31]–[33]. Additional approaches, such as targeted misexpression screens or maternal-effect mosaic screens have been applied to circumvent this limitation, but the number of genes acting cell autonomously in the PGCs is still very limited [22], [34]–[37]. Through an RNAi-based screening approach, however, we were able to eliminate both maternally provided as well as zygotic transcripts. This way, in addition to the recovery of known genes functioning in the PGCs (*Tre1*, *nos*, *pgc*), we identified several novel genes regulating various steps in germ cell development. In our experimental setup, we cannot discriminate between PGC-specific or somatic effects of the silenced genes. However, embryonic expression of many genes on our candidate list (*CG17658, CG14545, zpg*) is restricted to the PGCs representing potential candidates in the regulation of PGC development in a germ cell autonomous manner. Further experiments, such as genetic analysis of the loss of function mutant alleles and germ cell transplantation, will be necessary to individually analyze the germ cell-specific effect of these genes.

In our screen, the knock down of approximately 10% of the tested genes led to germ cell phenotype, which is very similar to the data obtained by RNAi-based functional transcriptome analysis in the planaria *Schmidtea mediterranea* [38]. A technical reason for this relatively low hit rate may be that RNAi is not efficient enough to eliminate all mRNAs of these genes. Application of a higher dsRNA concentration for microinjections could increase the number of identified genes [39]. To our experience, increasing the dsRNA concentration resulted in pleiotropic phenotypes, which made the phenotypic analysis ambiguous (data not shown). Therefore, we utilized a relatively low concentration of dsRNAs that resulted in a low hit rate but reliable phenotypic outcome. Another explanation for the low hit rate might be the functional redundancy in the PGC transcriptome. Our results do not support this interpretation, since we observed a very low rate of synthetic phenotypes during a systematic multiple RNAi experiment.

RNAi acts upstream of the protein level; therefore, maternally provided proteins were inaccessible by our screening strategy. As a consequence, known PGC regulator genes with high maternal protein contribution (*shg*, *piwi*, *vas*, *osk* and *pum*) were not recovered in our screen. Remarkably, there is no substantial overlap between the maternal transcriptome and proteome [2]. Additional approaches, such as inducible protein degradation techniques, could be applied to deplete maternally provided proteins and analyze their PGC-specific function [40], [41].

In summary, our screen provides a list of 48 germ line regulator genes most of which have not been implicated previously in germ cell development. Detailed analysis of these genes could contribute to a better understanding of multiple aspects of early germ cell development, such as PGC division, PGC migration, soma-germ line interactions or maintenance of the germ line.

### Co-RNAi revealed genetic interaction of *trio* and *Gap1* during germ cell development

Application of sensitized genetic backgrounds has been shown to be a powerful tool to identify dominant genetic interactions between germ line regulators [42]. Here we used a sensitized background which reduces the number of PGCs and reveals the involvement of the tested genes on germ line development. Dominant genetic interactions represent a strong indication for the role of the genes in germ cell development, but the interpretation of the interactions and further genetic analysis is difficult. To determine the exact role of the identified genes in germ cell development, it is necessary to perform an individual analysis of the phenotypes caused by loss of function mutant alleles.

Our genetic sensitization is based on the effect of mutant alleles of three genes and cannot be applied to test the interactions between arbitrarily chosen genes. Therefore, injection of pooled dsRNAs was used, which enables the easy, quick and simultaneous silencing of genes and large-scale detection of genetic interactions [43]–[45]. In this way, we found a genetic interaction between Rho and Ras regulator genes, the RhoGEF *trio* and the RasGAP *Gap1*, in regulating PGC development. In addition to their essential role in embryonic morphogenesis, Ras and Rho GTPases have been shown to be required for distinct steps PGC migration [22], [36], [46]. We propose that *trio* and *Gap1* regulate the activity of RhoA and Ras1, respectively, both in the PGCs and in the soma. RNAi-mediated partial inactivation of *trio* and *Gap1* individually does not have a detectable consequence on PGC development, but their simultaneous silencing cannot be tolerated by the embryo, leading to gonad formation defects.

Various developmental processes can compensate for genetic perturbations, resulting in robustness. Several mechanisms, such as functional redundancy between genes, networks of feedback and feedforward loops or microRNA mediated regulation of gene expression might contribute to phenotypic robustness [47]. In a systematic screen for genetic interactions, we found a low number of interacting gene pairs responsible for the regulation of PGC migration indicating a low degree of redundancy in the germ line transcriptome. The low level of functional overlap between genes suggests that additional mechanisms confer robustness to embryonic germ cell development. MicroRNA based gene silencing has been proposed to stabilize processes involved in the regulation of robustness of PGC migration in zebrafish and *Drosophila* [48], [49]. Our data provide indirect support for this hypothesis.

### RNAi screen revealed roles of pleiotropic genes *pbl* and *feo* in germ cell development

An additional difficulty of PGC analysis is the pleiotropic effect of the genes involved in germ cell development. RNAi provides an ideal method of overcoming this limitation, since by the application of RNAi a phenotypic series of various strengths is generated. In this way, we were able to detect a requirement of essential genes, such as *pbl* and *feo*, at different stages of germ line development.

*Pbl* acts in the embryo, whereas *feo* functions in the larval PGCs. Although *pbl* mRNA is enriched in germ cells, Pbl protein can be detected constitutively in the whole embryo [50]. *Pbl* has been shown to be involved in several processes required for PGC development, such as cell division and cell migration [51]–[53]. In the *pbl* mutants, the decreased number of PGCs and SGPs localize at abnormal positions, suggesting that *pbl* acts both in the germ line and in the SPGs. Alternatively, gonad formation defects may arise from abnormalities in additional developmental processes, relevant to germ line function, such as development of the mesoderm, midgut or nervous system [21], [54], [55]. In this case, all the PGC and SPG phenotypes are secondary effects of the problems in these tissues. We propose the hypothesis that a combined defect of several morphogenetic events leads to the detected complex germ cell migration phenotype in *pbl* mutants.

Feo belongs to the conserved Ase1/PRC1 family of microtubule crosslinker proteins [56]. In *Drosophila* neuroblasts, Feo localizes to the mitotic spindle midzone and is involved in mitosis by bundling the overlapping microtubule plus ends [57]. Similarly to the neuroblasts, Feo localizes to the mitotic spindle of the germ cells where it regulates the mitotic division. *Feo* is an essential gene and its loss-of-function alleles are lethal. Through dsRNA injection, we managed to establish a situation where reduction of *feo* activity still allows the animal to develop until adult age; however, germ cell division is impaired indicating that germ cells are more sensitive for the Feo expression level as somatic cells. In fact, increasing the concentration of the injected *feo* dsRNA leads to increased penetrance of the germ cell defects and lethality (data not shown).

### *Mei-P26*, a novel regulator of embryonic and larval germ cell survival

Mei-P26, a TRIM-NHL domain protein, is an important component of microRNA pathway mediated mRNA regulation [25]. It also has the capacity to directly associate with and repress translation of target mRNAs [26]. *Mei-P26* has been shown to be an essential regulator of germ line development. In addition to its critical function in germ line cyst differentiation, *mei-P26* has been shown to be required for GSC self-renewal during oogenesis [25], [26]. Since loss-of-function mutations of *mei-P26* cause female sterility, analysis of the *mei-P26* alleles by the maternal effect mosaic technique is unsuitable for revealing its function in the embryonic PGCs. RNAi, however, is an ideal method to study the requirement of *mei-P26* in PGC development. Here we present evidence that *mei-P26* is required for the maintenance of embryonic and larval PGCs.

We found that the embryonic phenotype of *mei-p26* silencing is similar to the phenotype of the maternal *nos* mutants, i.e. germ cell loss, PGC migration defects and rudimentary adult gonads [28]. Accordingly, Mei-P26 has been shown to physically interact with Nos and function as its translational co-repressors in the adult GSCs [26]. In other cellular contexts, Nos has been shown to form a complex with the Mei-P26 homolog TRIM-NHL protein Brain tumor (Brat) to repress the translation of target mRNAs, such as *CycB* [58]–[60]. In the PGCs, however, *brat* is dispensable for Nos mediated translational repression of *CycB* [58]. Therefore it has been proposed that in the PGCs, a spatially restricted co-repressor of Nos exists which regulates repression of *CycB* mRNA [61]. Here we show evidence that *mei-P26* is involved in the Nos-mediated translational repression of the cell cycle regulator *CycB* within the PGCs. We propose a model in which Mei-P26 interacts with Nos in the embryonic PGCs, ensuring PGC survival and inhibition of somatic differentiation by suppressing the translation of target mRNAs.

In the female larva, Mei-P26 might repress the translation of *nos*, similar to its role in differentiating adult cysts [64]. However, Mei-P26 mediated repression of *nos* translation takes place after germ cells have initiated differentiation. In these differentiating cells Mei-P26 associates with the germ line differentiation factor Bag of marbles (Bam) to repress *nos* translation. Wild type larval PGCs do not express Bam, therefore, it is unlikely that Mei-P26 would be required for translational repression of *nos* in the larval ovaries.

A possible function for Mei-P26 in larval ovaries could be that rather than repressing *nos* translation, it represses differentiation of PGCs by promoting Dpp signaling, similar to its role in adult GSCs [26]. In fact, Dpp signaling is active in the germ line throughout development and is required for the maintenance of PGC and GSC fate and for repression of their differentiation [11], [62], [63]. However, data obtained by the analysis of the larval *mei*-*P26* loss-of-function phenotypes do not support this hypothesis. Dpp signaling remains active in *mei-P26*-deficient larval ovaries, as indicated by the presence of pMad in the PGCs. This Dpp activity prevents PGCs from differentiating into fusome-containing germ line cysts. Therefore, we propose that Dpp mediated suppression of PGC differentiation is independent of *mei-P26* function.

A possible function for *mei-P26* in larval ovaries might be that rather than inhibiting Dpp dependent differentiation, it controls maintenance of the PGCs. In larval ovaries, proliferation of PGCs is regulated by a feedback interaction between somatic intermingled cells (ICs) and PGCs [6]. PGCs promote IC survival by activating the EGF signal transduction pathway in the ICs. In turn, ICs activate mechanisms in the PGCs, leading to a proliferation arrest. *Mei-P26* might be involved in coordinating these signals in the larval PGCs. Intriguingly, *mei-P26* has been proposed to specifically regulate the translation of various target mRNAs in a developmental context and cell type-dependent manner [26], [64]. Identification of novel binding partners and target mRNAs of Mei-P26 will elucidate its role in PGC development.

## Materials and Methods

### Drosophila stocks

Mutant alleles of the identified genes were provided by the Bloomington Drosophila Stock Center, Kyoto Stock Center, Szeged Stock Center and the Exelixis Collection (Table S7). For inducible silencing of the selected genes, UAS-RNAi and *nos-Gal4-VP16* lines were obtained from the Bloomington Drosophila Stock Center (Table S4). The *nos-Moe.EGFP.nos3'UTR* fly stock was provided by R. Lehmann.

### Embryo injection, RNAi screening and time-lapse analysis

Embryo collection and microinjection were performed as described earlier [65]. To silence the selected genes, the Heidelberg2 (BKN) RNAi library was used [20]. New dsRNAs targeting a different region of the 48 identified genes were generated by *in vitro* transcription (T7 RiboMAX Express, Promega) (Table S3). The concentration of injected dsRNA solution was ≈1 µg/µl in TE buffer. For the large-scale screen, after injection, the embryos were permitted to develop to stage 9 (4 hours after egg laying) under Voltalef H10S halocarbon oil (VWR) and were subsequently imaged at 25°C on an Olympus CellR fluorescent microscope. A 5X objective lens and an F-View II camera (Soft Imaging System, Münster) were used for time-lapse imaging. Embryo development was recorded for 13 hours, and images were acquired every 10 minutes. Time-lapse movies were analyzed using ImageJ software. In each independent experiment, 60 embryos were injected and analyzed; embryos leaking cytoplasm or exhibiting obvious patterning defects were ignored. If the penetrance of the given germ cell phenotype exceeded twice of the control in ¾ of the independent experiments, the gene was considered to be a positive hit.

For the co-silencing experiment, paralogous pairs were selected using the Ensemble 5.2 database. To select pairs based on overall sequence similarity, combinatorial multiple sequence alignment was performed by ClustalW and the similarity score of 15 was used as a cutoff. Gene pairs sharing identical functional domains were selected by the Uniprot 14.7 database. For simultaneous silencing of gene pairs, the concentration of each dsRNA was ∼1 µg/µl in TE buffer. In the single gene silencing control experiment, each dsRNA was extended with dsRNA specific to the indifferent Drosophila *galectin* gene to a total dsRNA concentration of ∼2 µg/µl. Significance values were determined by the Student's T-test. The dsRNA samples were coded, and injections of dsRNAs and analysis of the movies were performed blind.

### Germ-line specific silencing by inducible transgenic RNAi

For germ-line specific silencing, the *nos*-*Gal4-VP16* driver was used to express shRNAs targeting the identified genes. To silence the zygotic expression of the genes, *nos-Gal4-VP16/UAS-shRNA* flies were generated and screened for rudimentary gonads. To silence the genes of interest maternally, *nos-Gal4-VP16/UAS-shRNA* females were crossed to wild-type males and the progenies were screened for the germ cell-less phenotype.

### Dominant genetic interaction with a sensitized genetic background

Analysis of dominant genetic interactions of the identified genes was performed as described earlier [23]. Females bearing one copy of the *staufen^D3^*, *oskar^54^* and *tropomiosinII* ^eg9^ alleles and one copy of a loss of function allele of the gene of interest, were crossed to wild-type males and the progenies were scored for germ cell-less gonads. When the penetrance of the germ cell-less phenotype exceeded 20.7% (twice that of the control), the allele was considered to have interacted dominantly with the sensitized genetic background.

### Immunohistochemistry

Immunostainings of embryos were performed as described earlier [66]. For immunostaining of the larval gonads, the dissected ovaries and testes were fixed in 4% formaldehyde in PBS and rinsed three times with methanol. Fixed gonads were washed in PBS containing 1% Triton X-100 (PBT) for one hour and treated with primary antibodies diluted in PBT containing 1% bovine serum albumin and 5% fetal calf serum. Primary antibodies used were mouse anti CyclinB (1∶4, F2F4, DSHB), mouse anti-α-Tubulin (1∶100, Sigma), mouse anti-γ-Tubulin (1∶50, Sigma), rabbit anti-Mei-P26 (1∶200), mouse anti-Fas3 (1∶25, 7G10, DSHB), guinea-pig anti-Tj (1∶3000) [67], rat anti-vasa (1∶300, DSHB), goat anti-vasa (1∶150, Santa Cruz), mouse anti-Hts (1∶20, 1B1, DSHB), rabbit-anti-Feo (1∶100) [57], and rabbit anti-pSmad (1∶100, 41D10, Cell Signaling Technology). As secondary antibodies, an Alexa 647-conjugated anti-rat IgG (1∶600, Molecular Probes), Alexa 546-conjugated anti-rabbit and anti-guinea pig IgGs (1∶600, Molecular Probes), and an Alexa 488-conjugated anti-mouse IgG (1∶600, Molecular Probes), were used. Specimens were mounted in 50% glycerol/PBS and observed under an Olympus FW1000 confocal microscope. Z-stacks of optical sections were recorded, maximum intensity projections of the optical sections were made with ImageJ 1.46r (Wayne Rasband, NIH, USA), and intensity values and color balance were adjusted using the GIMP software (Ver.2.8.2).

## Supporting Information

Figure S1 **Immunofluorescence images of adult ovarioles.** Vasa staining labels germ cells (red), DAPI visualizes the nuclei (green). (A) Wild-type ovariole. (B–G) Expression of shRNAs in the germ line driven by the *nos-Gal4-VP16* driver induces rudimentary ovarioles with partial or complete loss of germ cells. (H) Expression of *hang*-*shRNAs* in the germ line induces the formation of cysts with abnormal germ cell number. Scale bars represent 20 µm.
(TIF)

Table S1 Expression pattern and BKN ID of genes tested by dsRNA microinjection.
(XLSX)

Table S2 Penetrance of the germ cell defects induced by dsRNA injection in the separate experiments. Yellow background indicates positive hits. In experiments 1.1 and 1.2, the Heidelberg2 (BKN) RNAi library was used. In experiment 2.1 and 2.2, dsRNAs targeting other regions of the 48 newly identified genes were used.
(XLSX)

Table S3 Primer sequences used to generate dsRNAs targeting other regions of the identified genes.
(XLSX)

Table S4 Penetrance of the adult germ cell defects induced by germ line specific expression of shRNAs.
(XLSX)

Table S5 Germ line specific functions of the identified *Drosophila* genes and their orthologs in other species.
(PDF)

Table S6 Gene pairs subjected to co-RNAi.
(XLSX)

Table S7 Dominant genetic interaction of the identified genes with the sensitized genetic background.
(XLSX)

Movie S1 Abnormal germ cell development generated by RNAi. Movies show abnormal germ cell development of dsRNA-injected embryos expressing Moesin:EGFP in the germ cells. Scale bar represents 50 µm.
(AVI)

Movie S2 **Three dimensional reconstruction of ovaries of third-stage larvae immunostained with anti-Vasa (red), anti-Tj (blue) and anti-Fas3 (green) antibodies.**
(AVI)
