# Supplementary figures and images for: Functional Analysis of the Drosophila Embryonic Germ Cell Transcriptome by RNA Interference
Source: PLoS One. 2014 Jun 4;9(6):e98579. doi: 10.1371/journal.pone.0098579 (PMC4045815; doi:10.1371/journal.pone.0098579)

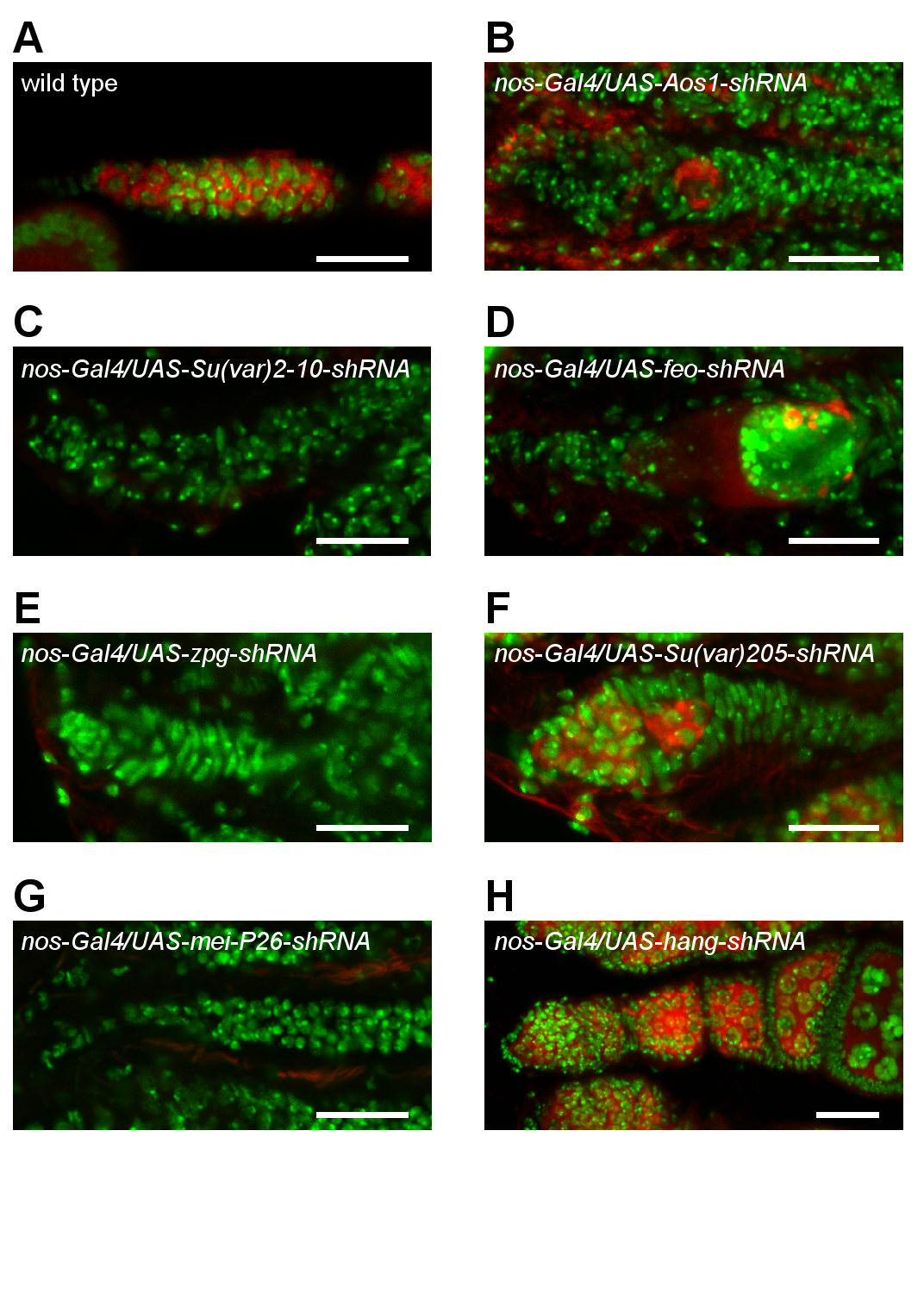

Supplement: Figure S1 — Immunofluorescence images of adult ovarioles. Vasa staining labels germ cells (red), DAPI visualizes the nuclei (green). (A) Wild-type ovariole. (B–G) Expression of shRNAs in the germ line driven by the nos-Gal4-VP16 driver induces rudimentary ovarioles with partial or complete loss of germ cells. (H) Expression of hang-shRNAs in the germ line induces the formation of cysts with abnormal germ cell number. Scale bars represent 20 µm. (TIF) [file pone.0098579.s001.tif]
