# Supplementary material for: Functional Analysis of the Drosophila Embryonic Germ Cell Transcriptome by RNA Interference
Source: PLoS One. 2014 Jun 4;9(6):e98579. doi: 10.1371/journal.pone.0098579 (PMC4045815; doi:10.1371/journal.pone.0098579)
Supplement: Table S5 — Germ line specific functions of the identified Drosophila genes and their orthologs in other species. (PDF) [file pone.0098579.s006.pdf]

| **Gene** | **Known function in germ cell development in Drosophila** | **Orthologs** | **Known funtion of orthologs in germ cell development** |
|---|---|---|---|
| CycA | maintenance of GSC[1]<br>Cystocyte cell cycle[2] | Ccna1; Ccna2(mouse) | Male meiosis [3,4]<br>Suggested role in female meiosis [5] |
| CycB | PGC and GSC proliferation [6] | cyb-1 (C.elegans)<br>ccnb1 (D. rerio)<br>Ccnb1 (mouse) | Meiosis [7]<br>Oocyte maturation [8]<br>Meiosis [9] |
| dsx | Sex determination [10] | dmrt genes | Sex determination [11] |
| nos | Germline formation [12]<br>GSC maintenance [13]<br>GSC differentiation [14] | nanos genes | Germ cell development [15–18] |
| ovo | Germ line sex determination [19]<br>Female germ line development [20] | Ovol1; Ovol2 (mouse) | Spermatogenezis [21,22] |
| pbl | Male meiosis [23] | Ect2 (mouse) | Oocyte meiosis [24] |
| Su(var)205 | - | Cbx1; Cbx3 (mouse) | Spermatogenesis [25]<br>PGC cell cycle [26] |
| orb | localization and transcription of osk mRNA [27,28]<br>Spermatogenezis [29] | CPEB (Xenopus) | Oocyte maturation [30,31] |
| Phf7 | Sex determination [32] | NYD-SP6/Phf7 (mouse) | Spermatogenezis [33] |
| bel | Germ line viability [34] | DDXY (human) | Spermatogenezis [35,36] |
| nclb | GSC maintenance in males [37] | - | - |
| spir | osk localization in oocyte [38] | Spire1, Spire2 (mouse) | Meiosis [39] |
| neur | GSC maintenance [40] | - | - |
| mael | Oocyte development [41,42]<br>GSC differentiation [43] | - | - |
| pgc | PGC development [44,45] | - | - |
| zpg | Germ cell differentiation [46,47] | inx-13 (C.elegans) | Oocyte maturation [48] |
| mei-P26 | Germ cell differentiation [49–51] | - | - |
| Tre1 | Germ cell migration [52] | - | - |
| scra | Spermatocyte development [53,54] | ani-1, ani-2 (C.elegans) | Ooocyte maturation, meiosis [55] |

## Supplementary References


1. Chen D, Wang Q, Huang H, Xia L, Jiang X, et al. (2009) Effete-mediated degradation of Cyclin A is essential for the maintenance of germline stem cells in Drosophila. Development 136: 4133–4142. doi:10.1242/dev.039032.
2. Lilly MA, de Cuevas M, Spradling AC (2000) Cyclin A associates with the fusome during germline cyst formation in the Drosophila ovary. Dev Biol 218: 53–63. doi:10.1006/dbio.1999.9570.
3. Liu D, Matzuk MM, Sung WK, Guo Q, Wang P, et al. (1998) Cyclin A1 is required for meiosis in the male mouse. Nat Genet 20: 377–380. doi:10.1038/3855.
4. Wolgemuth DJ, Lele KM, Jobanputra V, Salazar G (2004) The A-type cyclins and the meiotic cell cycle in mammalian male germ cells. Int J Androl 27: 192–199. doi:10.1111/j.1365-2605.2004.00480.x.
5. Persson JL, Zhang Q, Wang XY, Ravnik SE, Muhlrad S, et al. (2005) Distinct roles for the mammalian A-type cyclins during oogenesis. Reproduction 130: 411–422. doi:10.1530/rep.1.00719.
6. Wang Z, Lin H (2005) The division of Drosophila germline stem cells and their precursors requires a specific cyclin. Curr Biol 15: 328–333. doi:10.1016/j.cub.2005.02.016.
7. Van der Voet M, Lorson MA, Srinivasan DG, Bennett KL, van den Heuvel S (2009) C. elegans mitotic cyclins have distinct as well as overlapping functions in chromosome segregation. Cell Cycle 8: 4091–4102.
8. Kondo T, Yanagawa T, Yoshida N, Yamashita M (1997) Introduction of cyclin B induces activation of the maturation-promoting factor and breakdown of germinal vesicle in growing zebrafish oocytes unresponsive to the maturation-inducing hormone. Dev Biol 190: 142–152. doi:10.1006/dbio.1997.8673.
9. Polanski Z, Ledan E, Brunet S, Louvet S, Verlhac MH, et al. (1998) Cyclin synthesis controls the progression of meiotic maturation in mouse oocytes. Development 125: 4989–4997.
10. Shukla JN, Nagaraju J (2010) Doublesex: a conserved downstream gene controlled by diverse upstream regulators. J Genet 89: 341–356.
11. Kopp A (2012) Dmrt genes in the development and evolution of sexual dimorphism. Trends Genet 28: 175–184. doi:10.1016/j.tig.2012.02.002.

12. Kobayashi S, Yamada M, Asaoka M, Kitamura T (1996) Essential role of the posterior morphogen nanos for germline development in Drosophila. Nature 380: 708–711. doi:10.1038/380708a0.

13. Bhat KM (1999) The posterior determinant gene nanos is required for the maintenance of the adult germline stem cells during Drosophila oogenesis. Genetics 151: 1479–1492.

14. Forbes A, Lehmann R (1998) Nanos and Pumilio have critical roles in the development and function of Drosophila germline stem cells. Development 125: 679–690.

15. Köprunner M, Thisse C, Thisse B, Raz E (2001) A zebrafish nanos-related gene is essential for the development of primordial germ cells. Genes Dev 15: 2877–2885. doi:10.1101/gad.212401.

16. Tsuda M, Sasaoka Y, Kiso M, Abe K, Haraguchi S, et al. (2003) Conserved role of nanos proteins in germ cell development. Science 301: 1239–1241. doi:10.1126/science.1085222.

17. Julaton VTA, Reijo Pera RA (2011) NANOS3 function in human germ cell development. Hum Mol Genet 20: 2238–2250. doi:10.1093/hmg/ddr114.

18. Lai F, Singh A, King ML (2012) Xenopus Nanos1 is required to prevent endoderm gene expression and apoptosis in primordial germ cells. Development 139: 1476–1486. doi:10.1242/dev.079608.

19. Oliver B, Pauli D, Mahowald AP (1990) Genetic evidence that the ovo locus is involved in Drosophila germ line sex determination. Genetics 125: 535–550.

20. Mével-Ninio M, Terracol R, Kafatos FC (1991) The ovo gene of Drosophila encodes a zinc finger protein required for female germ line development. EMBO J 10: 2259–2266.

21. Dai X, Schonbaum C, Degenstein L, Bai W, Mahowald A, et al. (1998) The ovo gene required for cuticle formation and oogenesis in flies is involved in hair formation and spermatogenesis in mice. Genes Dev 12: 3452–3463.

22. Li B, Dai Q, Li L, Nair M, Mackay DR, et al. (2002) Ovol2, a mammalian homolog of Drosophila ovo: gene structure, chromosomal mapping, and aberrant expression in blind-sterile mice. Genomics 80: 319–325.

23. Giansanti MG, Farkas RM, Bonaccorsi S, Lindsley DL, Wakimoto BT, et al. (2004) Genetic dissection of meiotic cytokinesis in Drosophila males. Mol Biol Cell 15: 2509–2522. doi:10.1091/mbc.E03-08-0603.

24. Elbaz J, Reizel Y, Nevo N, Galiani D, Dekel N (2010) Epithelial cell transforming protein 2 (ECT2) depletion blocks polar body extrusion and generates mouse oocytes containing two metaphase II spindles. Endocrinology 151: 755–765. doi:10.1210/en.2009-0830.

25. Abe K, Naruse C, Kato T, Nishiuchi T, Saitou M, et al. (2011) Loss of heterochromatin protein 1 gamma reduces the number of primordial germ cells via impaired cell cycle progression in mice. Biol Reprod 85: 1013–1024. doi:10.1095/biolreprod.111.091512.

26. Brown JP, Bullwinkel J, Baron-Lühr B, Billur M, Schneider P, et al. (2010) HP1gamma function is required for male germ cell survival and spermatogenesis. Epigenetics Chromatin 3: 9. doi:10.1186/1756-8935-3-9.

27. Chang JS, Tan L, Schedl P (1999) The Drosophila CPEB homolog, orb, is required for oskar protein expression in oocytes. Dev Biol 215: 91–106. doi:10.1006/dbio.1999.9444.

28. Castagnetti S, Ephrussi A (2003) Orb and a long poly(A) tail are required for efficient oskar translation at the posterior pole of the Drosophila oocyte. Development 130: 835–843.

29. Xu S, Hafer N, Agunwamba B, Schedl P (2012) The CPEB protein Orb2 has multiple functions during spermatogenesis in Drosophila melanogaster. PLoS Genet 8: e1003079. doi:10.1371/journal.pgen.1003079.

30. Hake LE, Richter JD (1994) CPEB is a specificity factor that mediates cytoplasmic polyadenylation during Xenopus oocyte maturation. Cell 79: 617–627.

31. Standart N, Minshall N (2008) Translational control in early development: CPEB, P-bodies and germinal granules. Biochem Soc Trans 36: 671–676. doi:10.1042/BST0360671.

32. Yang SY, Baxter EM, Van Doren M (2012) Phf7 controls male sex determination in the Drosophila germline. Dev Cell 22: 1041–1051. doi:10.1016/j.devcel.2012.04.013.

33. Xiao J, Xu M, Li J, Chang Chan H, Lin M, et al. (2002) NYD-SP6, a novel gene potentially involved in regulating testicular development/spermatogenesis. Biochem Biophys Res Commun 291: 101–110. doi:10.1006/bbrc.2002.6396.

34. Johnstone O, Deuring R, Bock R, Linder P, Fuller MT, et al. (2005) Belle is a Drosophila DEAD-box protein required for viability and in the germ line. Dev Biol 277: 92–101. doi:10.1016/j.ydbio.2004.09.009.

35. Foresta C, Ferlin A, Moro E (2000) Deletion and expression analysis of AZFa genes on the human Y chromosome revealed a major role for DBY in male infertility. Hum Mol Genet 9: 1161–1169.

36. Ditton HJ, Zimmer J, Kamp C, Rajpert-De Meyts E, Vogt PH (2004) The AZFa gene DBY (DDX3Y) is widely transcribed but the protein is limited to the male germ cells by translation control. Hum Mol Genet 13: 2333–2341. doi:10.1093/hmg/ddh240.

37. Casper AL, Baxter K, Van Doren M (2011) no child left behind encodes a novel chromatin factor required for germline stem cell maintenance in males but not females. Development 138: 3357–3366. doi:10.1242/dev.067942.

38. Clark I, Giniger E, Ruohola-Baker H, Jan LY, Jan YN (1994) Transient posterior localization of a kinesin fusion protein reflects anteroposterior polarity of the Drosophila oocyte. Curr Biol 4: 289–300.

39. Pfender S, Kuznetsov V, Pleiser S, Kerkhoff E, Schuh M (2011) Spire-type actin nucleators cooperate with Formin-2 to drive asymmetric oocyte division. Curr Biol 21: 955–960. doi:10.1016/j.cub.2011.04.029.

40. Ward EJ, Shcherbata HR, Reynolds SH, Fischer KA, Hatfield SD, et al. (2006) Stem Cells Signal to the Niche through the Notch Pathway in the Drosophila Ovary. Current Biology 16: 2352–2358. doi:10.1016/j.cub.2006.10.022.

41. Clegg NJ, Frost DM, Larkin MK, Subrahmanyan L, Bryant Z, et al. (1997) maelstrom is required for an early step in the establishment of Drosophila oocyte polarity: posterior localization of grk mRNA. Development 124: 4661–4671.

42. Clegg NJ, Findley SD, Mahowald AP, Ruohola-Baker H (2001) Maelstrom is required to position the MTOC in stage 2-6 Drosophila oocytes. Dev Genes Evol 211: 44–48.

43. Pek JW, Lim AK, Kai T (2009) Drosophila maelstrom ensures proper germline stem cell lineage differentiation by repressing microRNA-7. Dev Cell 17: 417–424. doi:10.1016/j.devcel.2009.07.017.

44. Nakamura A, Amikura R, Mukai M, Kobayashi S, Lasko PF (1996) Requirement for a noncoding RNA in Drosophila polar granules for germ cell establishment. Science 274: 2075–2079.

45. Deshpande G, Calhoun G, Schedl P (2004) Overlapping mechanisms function to establish transcriptional quiescence in the embryonic Drosophila germline. Development 131: 1247–1257. doi:10.1242/dev.01004.

46. Tazuke SI, Schulz C, Gilboa L, Fogarty M, Mahowald AP, et al. (2002) A germline-specific gap junction protein required for survival of differentiating early germ cells. Development 129: 2529–2539.

47. Gilboa L, Forbes A, Tazuke SI, Fuller MT, Lehmann R (2003) Germ line stem cell differentiation in Drosophila requires gap junctions and proceeds via an intermediate state. Development 130: 6625–6634. doi:10.1242/dev.00853.

48. Whitten SJ, Miller MA (2007) The role of gap junctions in Caenorhabditis elegans oocyte maturation and fertilization. Dev Biol 301: 432–446. doi:10.1016/j.ydbio.2006.08.038.

49. Page SL, McKim KS, Deneen B, Van Hook TL, Hawley RS (2000) Genetic studies of mei-P26 reveal a link between the processes that control germ cell proliferation in both sexes and those that control meiotic exchange in Drosophila. Genetics 155: 1757–1772.

50. Insco ML, Bailey AS, Kim J, Olivares GH, Wapinski OL, et al. (2012) A self-limiting switch based on translational control regulates the transition from proliferation to differentiation in an adult stem cell lineage. Cell Stem Cell 11: 689–700. doi:10.1016/j.stem.2012.08.012.

51. Li Y, Maines JZ, Tastan OY, McKearin DM, Buszczak M (2012) Mei-P26 regulates the maintenance of ovarian germline stem cells by promoting BMP signaling. Development 139: 1547–1556. doi:10.1242/dev.077412.

52. Kunwar PS, Starz-Gaiano M, Bainton RJ, Heberlein U, Lehmann R (2003) Tre1, a G protein-coupled receptor, directs transepithelial migration of Drosophila germ cells. PLoS Biol 1: E80. doi:10.1371/journal.pbio.0000080.

53. Hime GR, Brill JA, Fuller MT (1996) Assembly of ring canals in the male germ line from structural components of the contractile ring. J Cell Sci 109 ( Pt 12): 2779–2788.

54. Goldbach P, Wong R, Beise N, Sarpal R, Trimble WS, et al. (2010) Stabilization of the actomyosin ring enables spermatocyte cytokinesis in Drosophila. Mol Biol Cell 21: 1482–1493. doi:10.1091/mbc.E09-08-0714.

55. Maddox AS, Habermann B, Desai A, Oegema K (2005) Distinct roles for two C. elegans anillins in the gonad and early embryo. Development 132: 2837–2848. doi:10.1242/dev.01828.
